# Changes in malaria epidemiology in a rural area of Cubal, Angola

**DOI:** 10.1186/s12936-014-0540-z

**Published:** 2015-01-21

**Authors:** Fernando Salvador, Yolima Cossio, Marta Riera, Adrián Sánchez-Montalvá, Cristina Bocanegra, Jacobo Mendioroz, Arlette N Eugenio, Elena Sulleiro, Warren Meredith, Teresa López, Milagros Moreno, Israel Molina

**Affiliations:** Department of Infectious Diseases, Vall d’Hebron University Hospital, Universitat Autònoma de Barcelona, Barcelona, Spain; PROSICS Barcelona, Barcelona, Spain; Department of Preventive Medicine and Epidemiology, Vall d’Hebron University Hospital, Barcelona, Spain; Hospital Nossa Senhora da Paz, Cubal, Angola; Department of Microbiology, Vall d’Hebron University Hospital, Barcelona, Spain; College of Agriculture and Environmental Sciences, University of South Africa, Pretoria, South Africa

**Keywords:** Malaria, Angola, Benguela, *Plasmodium falciparum*, Epidemiology

## Abstract

**Background:**

Scarce information about malaria epidemiology in Angola has been published. The objective of this study is to describe the epidemiology of malaria at the Hospital Nossa Senhora da Paz (Cubal, Angola) and the fatality rate due to malaria (total and in children under five years) in the last five years.

**Methods:**

A retrospective, observational study was performed at the Hospital Nossa Senhora da Paz, a 400-bed rural hospital located in Benguela Province of Angola. The study population included all patients who attended the hospital from January 2009 to December 2013. Outcome variables were calculated as follows: the percentage of malaria cases (number of positive thick blood films, divided by the total thick blood films performed); the percentage of in-patients for malaria (number of in-patients diagnosed with malaria, divided by the total number of in-patients); and, the fatality rate (number of deaths due to malaria divided by the number of positive thick blood films).

**Results:**

Overall, 23,106 thick blood films were performed, of which 3,279 (14.2%) were positive for *Plasmodium falciparum* infection. During this five-year period, a reduction of 40% (95% CI 37-43%, *p* < 0.001) in the malaria-positive slides was detected. Distribution of positive-malaria slides showed a seasonal distribution with a peak from December to March (rainy season). An average annual reduction of 52% (95% CI 50-54%, *p* < 0.001) in the admissions due to malaria was observed. The overall fatality rate due to malaria was 8.3%, and no significant differences in the annual fatality rate were found (*p* = 0.553).

**Conclusions:**

A reduction in the number of malaria cases and the number of admissions due to malaria has been observed at the Hospital Nossa Senhora da Paz, during the last five years, and incidence along the study period showed a seasonal distribution. All this information could be useful when deciding which malaria control strategies have to be implemented in this area.

## Background

Malaria is a major public health problem and one of the principal causes of morbidity and mortality in many African countries. Approximately half of all countries with ongoing malaria transmission are on track to meet the World Health Assembly and Roll Back Malaria target: to achieve a 75% reduction in malaria cases by 2015 compared to levels in 2000. Nevertheless, progress in more than a third of countries cannot be assessed due to limitations in their reported data. Unfortunately, surveillance systems are weaker where the malaria burden is higher [1].

Angola has emerged from three decades of civil war (1975-2002), which interrupted malaria control activities and severely damaged the public health infrastructure. Approximately 3.2 million cases of malaria were reported in 2004, of which two-thirds were in children under five years. *Plasmodium falciparum* is responsible for more than 90% of malaria infections in Angola and anopheline species most involved in transmission are *Anopheles gambiae*, *Anopheles funestus* and *Anopheles melas*. A national survey performed in 2011 showed malaria prevalence of 13% in children under the age of five. However, prevalence varies depending on the area: malaria is hyperendemic in the north, meso-endemic stable in the centre, and meso-endemic unstable in the south [2]. Apart from this survey, scarce information about malaria epidemiology has been published and where available, it only describes the northern region of Angola. Furthermore, this information shows malaria prevalence to be much lower in under-fives than reported previously [3,4].

**Figure 1:**
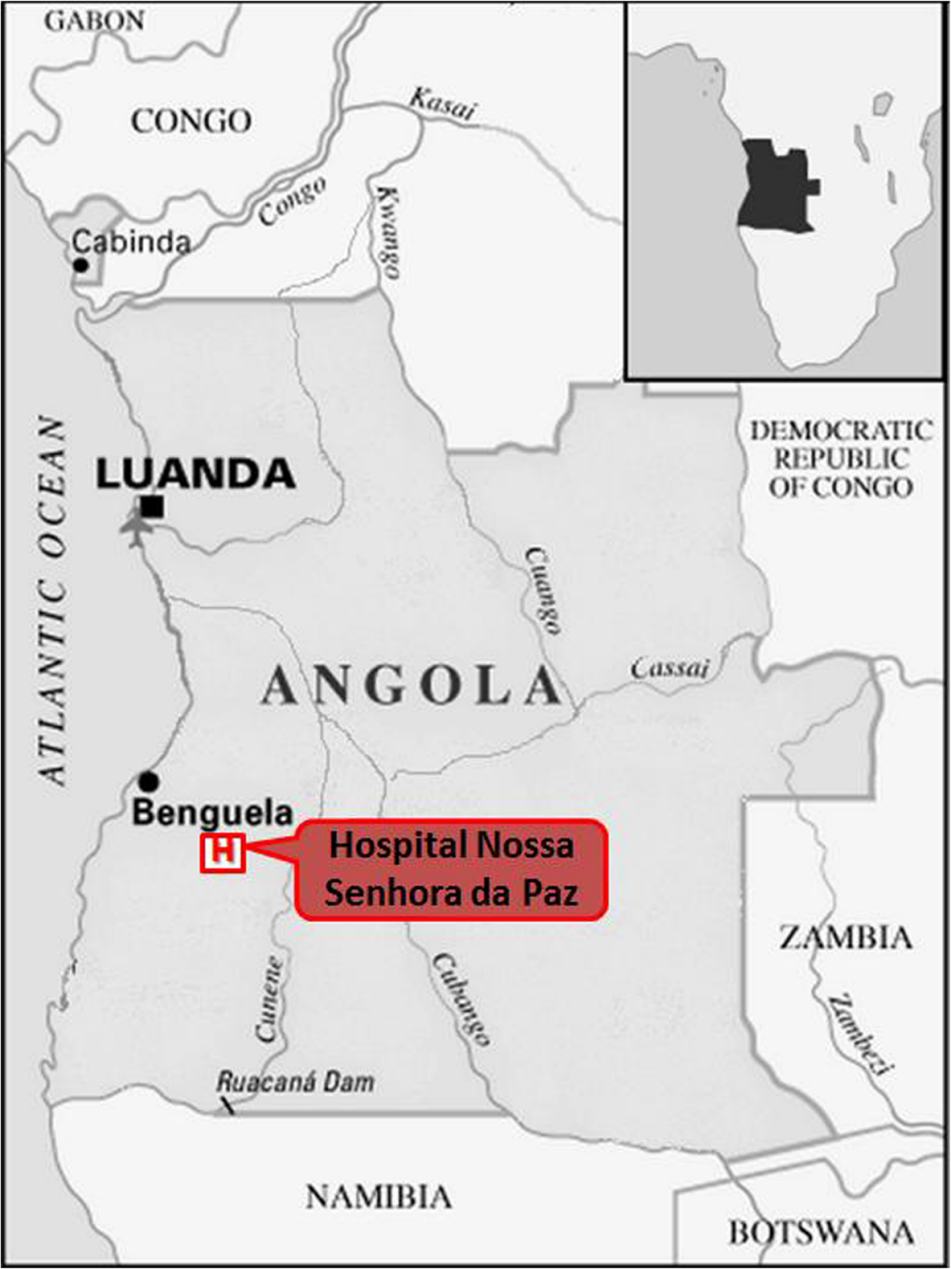
**Map of Angola.**

Cubal is a village of Benguela Province, located in western Angola, with a population of 300,000 inhabitants (Figure 1). The village is on the central highlands of Angola, 900 m above sea level and has a six-month wet season (from November to April). The malaria transmission in Cubal is reported to be meso-endemic stable according the national survey in 2011, however, there are no published data of the transmission in this area. The main health facility in Cubal is the Hospital Nossa Senhora da Paz (HNSP), and it is a referent for infectious pathology in the area. There is another hospital in Cubal (the Municipal Hospital), which also attends to patients with malaria. In 2007, an agreement was established with the Infectious Diseases Department of the Vall d’Hebron University Hospital (Spain), leading to various collaborative projects that focus mainly on malaria, soil-transmitted helminthes, tuberculosis and schistosomiasis. The objective of this study is to describe the epidemiology of malaria at the HNSP and the fatality rate due to malaria (total and in children under five years) in the last five years.

## Methods

### Study design

A retrospective observational study was conducted at the HNSP. This is a non-profit, 400-bed hospital that attends to, on average, 22,000 patients annually.

### Study population and data collection

The study population included all patients attending the HNSP from January 2009 to December 2013. Information was collected from hospital records, including in-patients’ records (total number of in-patients, age of in-patients, number of in-patients and deaths due to malaria, number of deaths for all causes), and laboratory records (total number of and positive thick blood films performed in all patients attending the facility, including in-patients and out-patients). It is important to note that in the in-patient registry, the definition of malaria case could include diagnosis of malaria based on clinical suspicion.

### Malaria clinical protocols

During the study period, a thick blood film examination (with Giemsa staining) was done routinely for all patients with fever attending the facility. Malaria was managed following the national guidelines of Angola: intravenous quinine was used as a first-line treatment for severe malaria, and oral quinine plus clindamycin (from 2009 to 2012), or artemether-lumefantrine (since 2012) as a first-line treatment for non-complicated malaria.

### Rainfall data

Information about rainfall in Cubal was obtained from World Weather Online [5]. This website has its own weather forecasting model which delivers reliable and accurate weather information for any geo-point in the world.

### Statistical analysis

Data from hospital and laboratory registries was double entered and validated into a Microsoft Excel Database, and later analysed using Stata (version 11.0). Outcome variables were calculated as follows: the percentage of malaria cases (number of positive thick blood films, divided by the total thick blood films performed); the percentage of in-patients for malaria (number of in-patients diagnosed with malaria, divided by the total number of in-patients); and, the fatality rate (number of deaths due to malaria divided by the number of positive thick blood films). All data were aggregated by month and year. Subanalysis for population under five years old was made. The Poisson regression was used to compare the yearly rate for each outcome variable. Results were considered statistically significant if the two-tailed P value was <0.05.

### Ethical considerations

The study protocol was approved by the institutional review board of the hospital. All data were anonymized and the confidentiality of the information was respected throughout the study, in accordance with the ethical standards of the Declaration of Helsinki.

## Results

**Figure 2:**
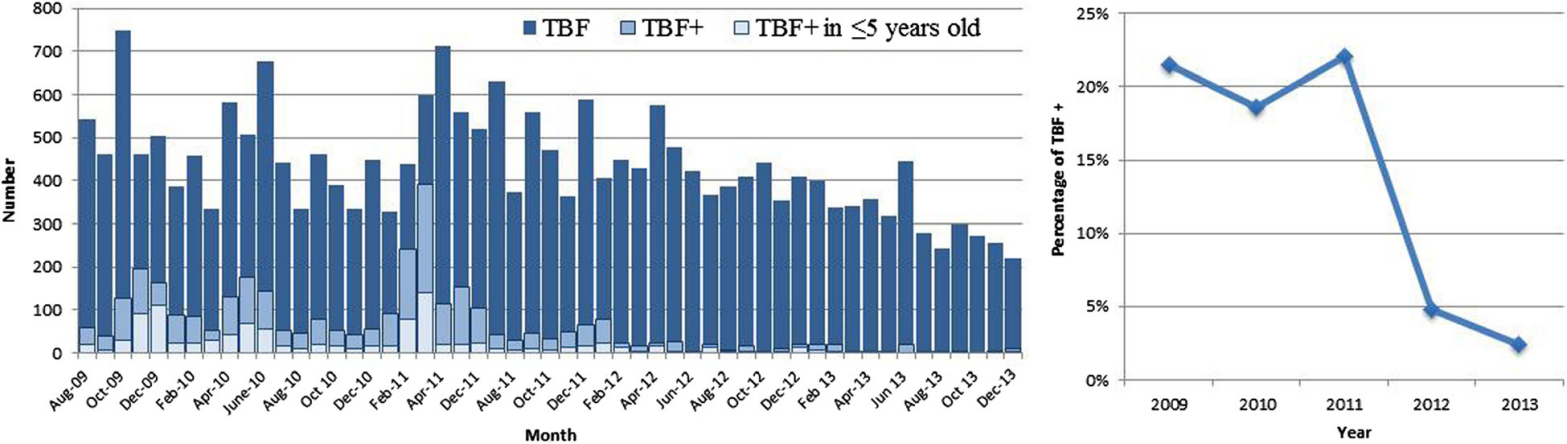
**Monthly number of malaria thick blood films (left panel) and yearly proportion of positive thick blood films (right panel) in Hospital Nossa Senhora da Paz.** TBF, thick blood films performed; TBF+, thick blood films positives.

**Figure 3:**
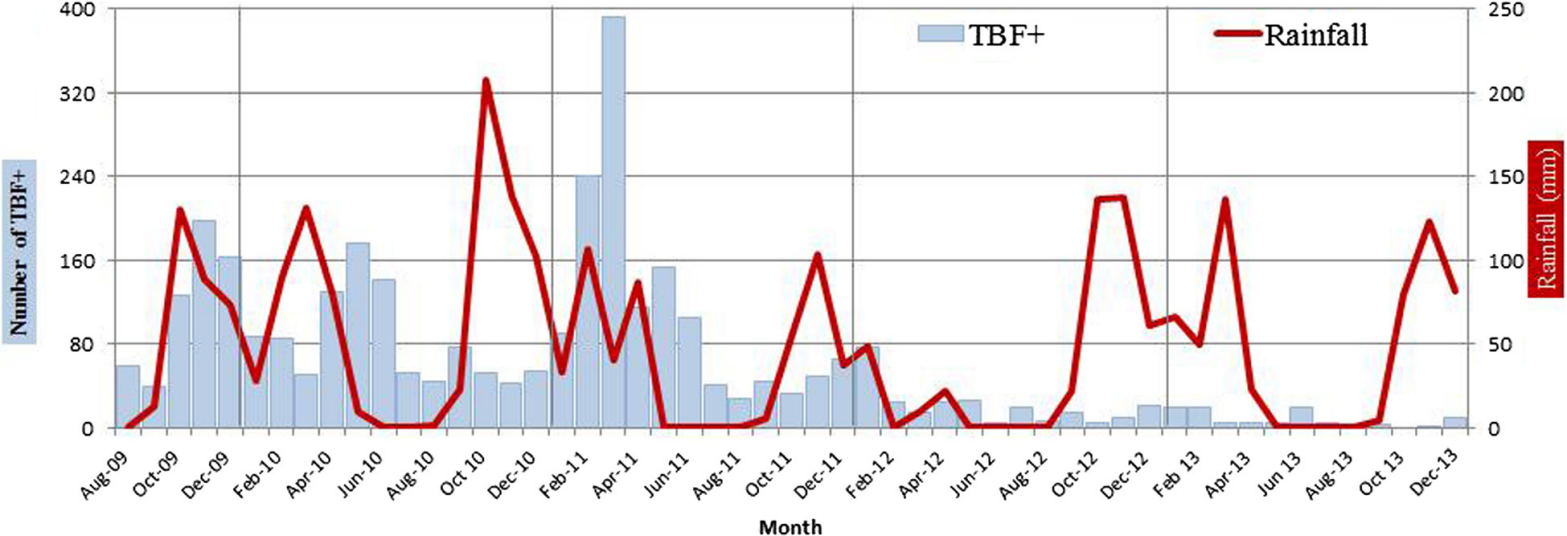
**Monthly number of malaria thick blood films in Hospital Nossa Senhora da Paz (blue columns), and monthly rainfall in Cubal.** TBF+, thick blood films positives.

Overall, 23,106 thick blood films were performed, of which 3,279 (14.2%) were positive for *P. falciparum* infection (data from laboratory were only available from August 2009). A reduction of 40% (95% CI 37-43%, *p* < 0.001) in the malaria-positive slides was observed during the study period (Figure 2). When describing the distribution of positive-malaria slides during the study period, a seasonal distribution was shown with a peak from December to March (wet season), and fewer cases during the driest months of the year. As Figure 3 shows, every increase in rainfall was followed by a peak in the malaria incidence; the only exception was the rainy season of 2012-2013, where malaria incidence was very low despite the increase in rainfall.

**Figure 4:**
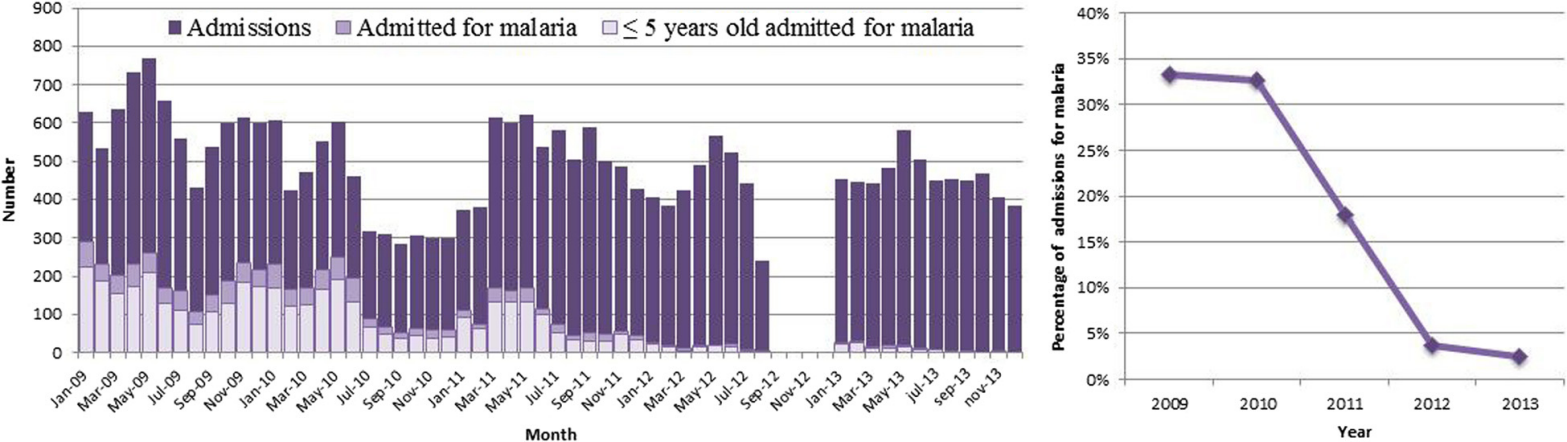
**Monthly number of hospital admissions (left panel) and yearly proportion of admissions for malaria (right panel) in Hospital Nossa Senhora da Paz.**

**Figure 5:**
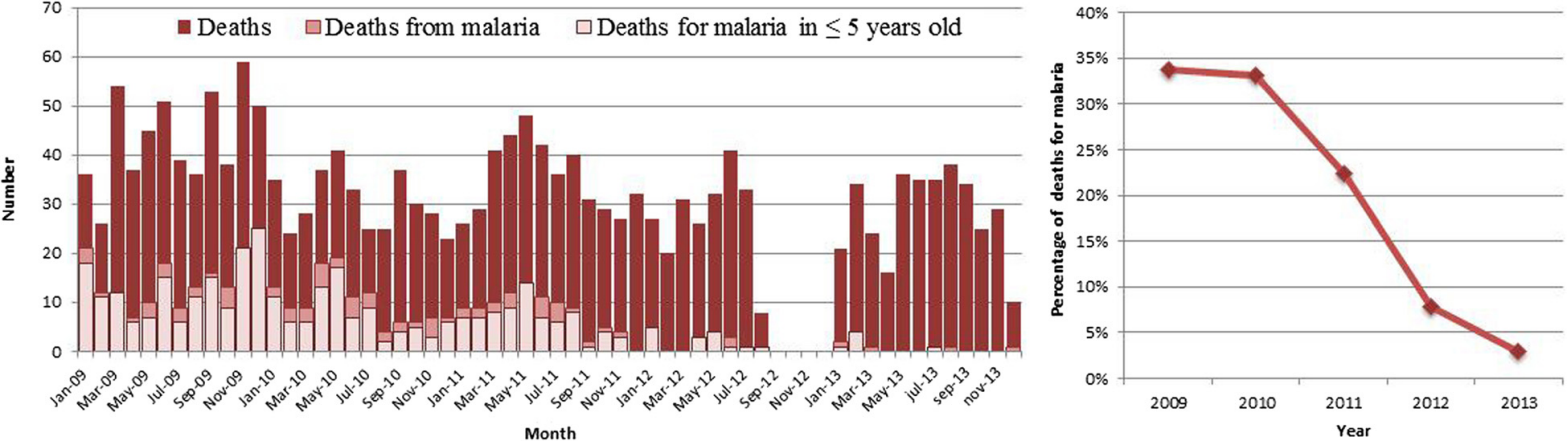
**Monthly number of hospital deaths (left panel) and yearly proportion of deaths for malaria (right panel) in Hospital Nossa Senhora da Paz.**

Taking into account the information from the in-patients registry, 27,452 patients were admitted to the hospital with a wide range of diagnosis during this five-year period,

An average annual reduction of 52% (95% CI 50-54%, *p* < 0.001) in the admissions due to malaria was detected (Figure 4). Of these in-patients, 1,870 died and 420 of these deaths were attributed to malaria; there was a reduction of 34% (95% CI 35-51%, *p* < 0.001) in the deaths attributed to malaria (Figure 5).

**Figure 6:**
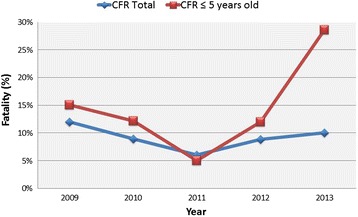
**Case fatality rate for malaria in Hospital Nossa Senhora da Paz.** CFR, case fatality rate.

The overall fatality rate due to malaria (number of deaths due to malaria divided by the number of positive thick blood films) was 8.3%. When comparing the annual fatality rate during the study period, no significant differences were found (*p* = 0.553) (Figure 6).

The population under five years old also had a reduction in the number of admissions due to malaria, the deaths attributed to malaria, and malaria-positive slides during the study period (Figures 2, 4 and 5). Nevertheless, when analysing the fatality rate in this population, there was an increase during the study period (Figure 6), although it was not statistically significant (*p* = 0.084).

## Discussion

In the present study, an important reduction in the number of malaria cases diagnosed in the HNSP during the last five years is observed, as well as in the number of admissions and deaths attributed to malaria. As mentioned previously, no data about local malaria prevalence have been reported in Angola, outside of the northern region. Therefore, this study provides new and valuable information about the current malaria prevalence in a previously non-evaluated area.

The reduction in the malaria prevalence has been observed not only in the northern part of Angola, but in other sub-Saharan countries, such as Senegal and The Gambia [3,4,6,7]. Possible reasons for the decrease of malaria in this area of Angola may include socio-economic changes, implementation of prevention measures against malaria and possible changes in environmental factors, such as rainfall. Angola was one of the first three countries selected as a President’s Malaria Initiative (PMI) target country in 2005. The PMI has supported activities in the country (in accordance with the National Malaria Control Programme) since this time, including universal coverage of long-lasting insecticide-treated nets (LLINs), indoor residual spraying (IRS), intermittent preventive treatment for pregnant women and improvement of case management through procurement of diagnostic equipment and treatment with artemisinin-based combination therapy (ACT). Nevertheless, the implementation of these activities has been irregular through the country; Benguela, for example, did not receive IRS, and ACT was scarcely available until 2012 [8].

The relationship between malaria and rainfall has been described for a long time, as malaria is a highly climate-sensitive, vector-borne, infectious disease [9-11]. Although information in this study about rainfall in Cubal was based on estimations, it showed a dramatic decrease in rainfall during the wet season of 2012 and incidentally, the reduction in malaria cases are more noticeable from this period. Different institutions have been alerted to rainfall changes in Benguela Province during recent years: the late start of the seasonal rains, irregular rainfall and dryness [12]. This reduction in rainfall in Benguela Province may be one of a host of environmental factors influencing the reduction in the number of malaria cases diagnosed in the HNSP during the study period. Other factors may involve changes in the temperature, in the occurrence of stagnant water bodies, and in the socio-economic conditions. Furthermore, the distribution of malaria cases along the study period is irregular; peaks in malaria incidence coincide with the wet season. Seasonal transmission has not been previously reported in this area, as it was thought to be a meso-endemic stable transmission area [2]. Seasonal transmission of malaria has important implications, both in clinical presentation of the disease and in the implementation of control strategies. The find of seasonal transmission in Cubal could be very relevant for considering seasonal chemoprevention for children, in the same way that it has been increasingly adopted in other areas with seasonal transmission [13,14]. Health public strategies that aim to eradicate malaria vary depending on the transmission pattern of the area, so all the information regarding this aspect is welcome and appreciated.

The distribution of malaria morbidity and mortality within communities depends directly upon transmission intensity; where the risk of infection is low, almost all exposed people are at substantial risk of severe disease [15]. Although the number of malaria cases diagnosed at the HNSP has decreased over the last five years, there were no changes in the overall fatality rate due to malaria. An increase in the fatality rate was observed only in children under five years old, but this was not statistically significant and was probably due to the scarce number of cases diagnosed in 2012-2013 within this population group. Different studies have addressed the relationship between clinical severity and the loss of immunity to malaria due to lower exposure to infective-mosquito bites, as happens in migrants to non-endemic countries or after implementation of reducing-exposure interventions (LLINs, IRS). However, this loss of population immunity to malaria seems to happen many years (sometimes decades) after reducing the exposure [16,17]. This gradual loss of immunity to malaria after the implementation of control strategies may explain the unchanged fatality rate of malaria in the present study, since it only covers five years of follow-up.

This study has some limitations derived from its retrospective condition; data were retrieved from medical records, the possibility of in-patients with malaria diagnosis based on clinical suspicion, and information from some months is missing (from January to July 2009 in the laboratory registry, and from September to December 2012 in the in-patients registry). Another issue is the lack of precise information about rainfall in Cubal that would allow confirmation of the relationship between peaks in malaria cases and increases in precipitation. Moreover, the study is performed only with data from a single hospital, which could be a limitation when interpreting the results, as it may not represent the situation in the whole community.

However, the study has strengths, such as the consistency between two different sources of information (laboratory registry and in-patients registry), which minimize some problems: missing information, hospitalization criteria, cases of malaria based on clinical suspicion. Another strong point of the study is that malaria slides were performed by the same, well-trained staff during the study period and continuously monitored laboratory procedures were applied.

## Conclusions

This is the first epidemiological study about malaria in this area of Benguela Province. During this five-year period, a reduction in the number of malaria cases, the number of admissions due to malaria and deaths attributed to malaria has been observed at the HNSP. The distribution of positive-malaria slides shows a seasonal distribution with a peak from December to March. Although the number of malaria cases has substantially decreased, no changes in the fatality rate have been observed during the study period. This information could be useful when deciding which malaria control strategies have to be implemented in this area. Prospective and multicentre studies must be supported in order to obtain more representative information on the Province.

## References

[1] WHO: World Malaria Report 2013. Available at: http://www.who.int/malaria/publications/world_malaria_report_2013/report/en/.

[2] Angola Malaria Indicator Survey 2011. 2012. Final Report. Available at: http://dhsprogram.com/pubs/pdf/MIS11/MIS11.pdf.

[3] Magalhães RJ, Langa A, Sousa-Figueiredo JC, Clements AC, Nery SV (2012). Finding malaria hot-spots in Northern Angola: the role of individual, household and environmental factors within a meso-endemic area. Malar J.

[4] Sousa-Figueiredo JC, Gamboa D, Pedro JM, Fancony C, Langa AJ, Magalhães RJ (2012). Epidemiology of malaria, schistosomiasis, geohelminths, anemia and malnutrition in the context of a demographic surveillance system in northern Angola. PLoS One.

[5] Cubal historical weather, Angola: World Weather Online. Available on: http://www.worldweatheronline.com/Cubal-weather-history/Benguela/AO.aspx. Last accessed on 1^st^ August 2014.

[6] Ceesay SJ, Casals-Pascual C, Erskine J, Anya SE, Duah NO, Fulford AJ (2008). Changes in malaria indices between 1999 and 2007 in The Gambia: a retrospective analysis. Lancet.

[7] Trape JF, Tall A, Sokhna C, Ly AB, Diagne N, Ndiath O (2014). The rise and fall of malaria in a west African rural community, Dielmo, Senegal, from 1999 to 2012: a 22 year longitudinal study. Lancet Infect Dis.

[8] President’s Malaria Initiative. Angola. Malaria Operational Plan FY 2014. Available on: http://www.pmi.gov/docs/default-source/default-document-library/malaria-operational-plans/fy14/angola_mop_fy14.pdf?sfvrsn=12. Last accessed on 1^st^ August 2014.

[9] Gao HW, Wang LP, Liang S, Liu YX, Tong SL, Wang JJ (2012). Change in rainfall drives malaria re-emergence in Anhui Province, China. PLoS One.

[10] Jusot JF, Alto O (2011). Short term effect of rainfall on suspected malaria episodes at Magaria, Niger: a time series study. Trans R Soc Trop Med Hyg.

[11] Yewhalaw D, Getachew Y, Tushune K, Michael KW, Kassahun W, Duchateau L (2013). The effect of dams and seasons on malaria incidence and anopheles abundance in Ethiopia. BMC Infect Dis.

[12] The Famine Early Warning System Network (FEWS NET). Available on: http://www.fews.net/southern-africa/angola/remote-monitoring-report/april-2014. Last accessed on 1^st^ August 2014.

[13] Guillebaud J, Mahamadou A, Zamanka H, Katzelma M, Arzika I, Ibrahim ML (2013). Epidemiology of malaria in an area of seasonal transmission in Niger and implications for the design of a seasonal malaria chemoprevention strategy. Malar J.

[14] Tine RC, Ndour CT, Faye B, Cairns M, Sylla K, Ndiaye JL (2014). Feasibility, safety and effectiveness of combining home based malaria management and seasonal malaria chemoprevention in children less than 10 years in Senegal: a cluster-randomised trial. Trans R Soc Trop Med Hyg.

[15] Doolan DL, Dobaño C, Baird JK (2009). Acquired immunity to malaria. Clin Microbiol Rev.

[16] Pistone T, Diallo A, Mechain M, Receveur MC, Malvy D (2014). Epidemiology of imported malaria give support to the hypothesis of “long-term” semi-immunity to malaria in sub-Saharan African migrants living in France. Travel Med Infect Dis.

[17] Ghani AC, Sutherland CJ, Riley EM, Drakeley CJ, Griffin JT, Gosling RD (2009). Loss of population levels of immunity to malaria as a result of exposure-reducing interventions: consequences for interpretation of disease trends. PLoS One.

